# Diagnostic and classification tools for chronic headache disorders: A systematic review

**DOI:** 10.1177/0333102418806864

**Published:** 2018-10-18

**Authors:** Rachel Potter, Katrin Probyn, Celia Bernstein, Tamar Pincus, Martin Underwood, Manjit Matharu

**Affiliations:** 1 Warwick Clinical Trials Unit, Warwick Medical School, University of Warwick, Coventry, UK; 2 Department of Psychology, Royal Holloway, University of London, Egham Hill, Egham, Surrey, UK; 3 Headache Group, Institute of Neurology and The National Hospital for Neurology and Neurosurgery, Queen Square, London, UK

**Keywords:** Chronic headache, primary care, diagnostic criteria

## Abstract

**Background or aim:**

Despite guidelines and the International Classification of Headache Disorders (ICHD-III beta) criteria, the diagnosis of common chronic headache disorders can be challenging for non-expert clinicians. The aim of the review was to identify headache classification tools that could be used by a non-expert clinician to classify common chronic disorders in primary care.

**Methods:**

We conducted a systematic literature review of studies validating diagnostic and classification headache tools published between Jan 1988 and June 2016 from key databases: MEDLINE, ASSIA, Embase, Web of Knowledge and PsycINFO. Quality assessment was assessed using items of the Quality of Diagnostic Accuracy Studies (QUADAS-2).

**Results:**

The search identified 38 papers reporting the validation of 30 tools designed to diagnose, classify or screen for headache disorders; nine for multiple headache types, and 21 for one headache type only. We did not identify a tool validated in a primary care that can be used by a non-expert clinician to classify common chronic headache disorders and screen for primary headaches other than migraine and tension-type headache in primary care.

**Conclusions:**

Despite the availability of many headache classification tools we propose the need for a tool that could support primary care clinicians in diagnosing and managing chronic headache disorders within primary care, and allow more targeted referral to headache specialists.

## Introduction

Around 4% of primary care consultations and 30% of neurology outpatient appointments in the UK are due to headache disorders (1,2).

Yet many patients presenting in primary care with headache do not have a formal diagnosis, are misdiagnosed, and can potentially receive inappropriate drug treatment and management (3). A study in the UK found 70% presenting with new onset headache were not formally diagnosed (3) and 88% of patients with a history of sinus headache screened in primary care clinics in the US met International Headache Society criteria for migraine (4).

Despite deceptively simple diagnostic criteria for different headache types such as the comprehensive headache classification of the International Classification of Headache Disorders, 3rd edition (5) and the National Institute for Health and Care Excellence (NICE) headache guidance (6); in reality, it can be challenging for a non-expert clinician to accurately diagnose headache disorders.

The Chronic Headache and Self-management Study (CHESS) is a National Institute for Health Research (NIHR) grant-funded programme (project number RP-PG-1212-20018) with the overall aim of developing and testing a self-management programme for people living with chronic headache. As part of the study we want to be able to classify participant's chronic headache types both for reporting purposes, and as part of the study intervention to allow targeted treatment and advice. Specifically, we need a classification tool that can be used by a non-expert clinician to screen for primary headache disorders other than migraine and tension type headache (TTH), distinguish between chronic migraine and chronic TTH and identify medication overuse headache (MOH) in primary care settings. We anticipate that such a tool could also support primary care clinicians in diagnosing and managing chronic headache disorders within primary care, and allow more targeted referral to headache specialists.

We therefore conducted this systematic review to a) identify any existing tools used to classify chronic headache disorders and b) assess the validation of tools identified.

## Methods

We registered this review prospectively with the International Prospective Register of Systematic Reviews. PROSPERO 2015: CRD42015019863 (7) and we followed the PRISMA guidelines for the reporting of systematic reviews (8).

### Search strategy and study selection

We included studies that describe the validation of headache tools intended to diagnose, classify or screen for one or more headache types and compare with a reference standard. We only included studies published in English and published from January 1988, the publication date of the first International Classification of Headache Disorders (ICHD) (5). We excluded studies with participants aged below 18 years and any dissertation and conference proceedings, plus those studies where the sole purpose of the study was to report the validation of a tool in a different language.

With the support of an academic support librarian, we searched key databases: MEDLINE, ASSIA, Embase, Web of Knowledge and PsycINFO. The searches were updated in June 2016. We used free text and MeSH terms based on those used for NICE headache guidelines 2012 (9); search words included: ‘Headache’, ‘headache disorders’, ‘headache disorders primary’, ‘headache disorders secondary’, ‘migraine’, ‘migraine disorders’, ‘migraine with aura’, ‘migraine without aura’, ‘tension headache’, ‘cluster headache’, ‘medication overuse headaches’ combined with ‘classification’, ‘diagnostic’, ‘diagnosis’, ‘diagnostic techniques and procedures’, ‘sensitivity’, specificity’, ‘efficacy’, ‘effectiveness’, ‘efficiency’, ‘predictive value of tests’, ‘likelihood function’, ‘diagnostic odds ratio’, and ‘screening,’ ‘questionnaire,’ ‘survey’, ‘interview as topic’ and ‘tools’, ‘instruments’ and ‘ICHD’.

Results were managed using the Evidence for Policy and Practice Information and Co-ordinating Centre (EPPI Centre) reviewer 4 software, duplicates removed, and titles checked for relevance by two authors (RP, KP). We sought a full copy of possible relevant papers, which were assessed independently for inclusion by two authors and any disagreement resolved by discussion. We checked reference lists of relevant papers for any additional studies.

### Data extraction and quality assessment

Three authors (RP, CB, KP) independently extracted data from papers included in the review using a predetermined pro-forma to capture both study and tool-specific information: Study design, characteristics of study participants, a brief description of the diagnostic or classification tool, test characteristics, diagnostic/classification parameters, validation assessment (sensitivity, specificity), how the tool is used (questionnaire, online, interview) and by whom (expert, non-expert, patient).

The same three authors independently assessed the methodological quality of each study using the Quality of Diagnostic Accuracy Studies (QUADAS-2), a validated tool used for the quality assessment of diagnostic accuracy studies (10).

The QUADAS-2 assesses four domains for risk of bias: Patient selection (sampling and exclusions), index test (conduct and interpretation), reference standard (conduct and interpretation), and flow and timing (interval between index and reference standard, number receiving reference standard and included in the analysis). The sets of signalling questions used for each domain have been tailored to the content of the review as recommended by the authors of the tool.

We assessed risk of bias as low, high or unclear for each domain and calculated an overall risk of bias dependent on the number of domains judged as high risk of bias: 0 domains = low, 1 = low/medium, 2 = medium, 3 = medium/high, 4 = high risk of bias. Where criteria used to judge risk for one of the domains was unclear, the risk was considered “high” and this was denoted in the overall risk of bias.

## Results

### Study selection

**Figure 1.**
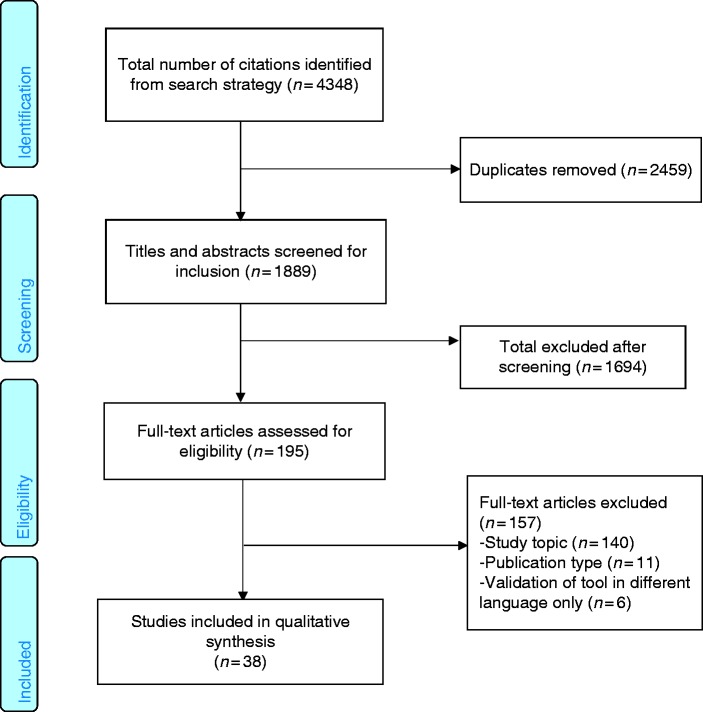
PRISMA flow chart of article selection.

We identified 4348 records from the combined database searches and removed 2459 duplicates. The remaining 1889 records were screened for relevance and 1694 records were excluded because they did not meet the study inclusion criteria. We obtained the full text for 195 records and excluded 157 after reading the full papers, which did not meet the inclusion criteria, resulting in a total of 38 papers included in the review (Figure 1).

### Study characteristics

The 38 papers published between 1991 and 2016 (11–48) report validation studies from 18 different countries, with most papers from the US (n = 10) and Italy (n = 4). The papers report the validation of 30 tools, nine to diagnose or classify more than one headache type and 21 to classify or screen for one headache type only: Migraine (n = 18), cluster headache (n = 2), and probable medication overuse headache (n = 1) (Table 1).

**Table 1.** Characteristics of included studies .

Name and brief description of tool	Type of tool (reported)	Author/year	Country	Study setting	Reference test	Psychometric results: Sensitivity (%), specificity (%), positive (PPV) value (%), negative predictive values (NPV) (%)
*Multiple headache types*						
AIDA Cefalee computer- assisted diagnostic expert system based on the ICHD-II to support diagnostic accuracy once all clinical characteristics are collected by medical staff. Headaches identified: Migraine (with aura, without aura, TTH, trigeminal autonomic cephalalgias	Diagnostic	De Simone R (2007) (14)	Italy	Clinical records of previously diagnosed primary headache cases from headache centres (n = 200)	Structured interview by medical staff working at headache centre using ICHD-II criteria	Overall sensitivity 98.5%
Computerised clinical decision support system (CDSS) based on ICHD-III beta to help community doctors, GPs and inexperienced physicians to simplify clinical diagnosis. Headaches identified: Migraine (with, without aura, chronic and probable) TTH (episodic, chronic, probable), cluster headache (probable), medication overuse headache and “others”	Diagnostic	Dong Z, et al. (2014) (15)	China	Patients from a headache centre (n = 543)	Headache specialists used the information entered into the CDSS to make their own “gold standard” diagnosis	Reported for each headache type. Sensitivity: 60.9–100 (low for probable migraine and probable TTH) Cluster headache 90 NDPH 100 Specificity: 97.9–100 PPV: 73.7–100 NPV: 95.6–100
Computerised Headache Assessment Tool (CHAT) online self-assessment reviewed with a doctor. Diagnosis based on IHS criteria to identify: episodic, probable and transformed migraine, new daily persistent headache, episodic and chronic tension-type headache, cluster headache, MOH.	Diagnostic	Maizels M and Wolfe J (2008) (35)	USA	Convenience sample of patients presenting with headache at an urgent care department, plus patients from a family practice waiting room (n = 117)	Telephone interview by headache specialist nurse based on validated diagnostic tools and IHS criteria	Sensitivity: Overall 88.9 (excluding MOH) Migraine 94.4 Daily headache 92.6 Medication overuse 82.7 Cluster headache 100 NDPH 42.9
Headache-Attributed Restriction, Disability, Social Handicap and Impaired Participation (HARDSHIP) questionnaire: Diagnosis of migraine, TTH or MOH generated by a computerised algorithm. The most bothersome headache type is diagnosed. Interview conducted by a trained non-medical interviewer	Diagnostic (module within questionnaire)	Ayzenberg I, et al. (2011) (12)	Russia	A sub-sample of respondents completing the questionnaire who had been randomly selected from four cities and three rural areas of Russia (n = 190)	Telephone interview by neurologist using expertise and ICHD-II criteria	Sensitivity: Migraine: 76.9 (68.1–84.0); TTH: 64.0 (57.9–68.4) Specificity: Migraine: 82.4 (77.8–86.1); TTH: 91.1 (85.6–94.9) PPV: Migraine: 69.4 (61.5–75.8); TTH: 86.4 (78.0–77.3) NPV: Migraine 87.3 (82.4–91.2) TTH 74.2 (69.8–77.3)
HARDSHIP questionnaire		Herekar A, et al. (2013) (25)	Pakistan	Consecutive sample recruited from three (urban and rural) medical sites (n = 180). Included patients reporting headache disorder and their attendants (i.e. non-patients)	Face-to-face interview using the ICHD-II criteria, conducted by a neurologist (expert in headaches)	Sensitivity: Migraine: 74; TTH 60; headache on more than 15 days: 98; probable MOH: 86 Specificity: Migraine: 87; TTH: 92; headache on more than 15 days: 100; probable MOH: 82 PPV: Migraine: 60; TTH: 69; headache on more than 15 days: 100; probable MOH: 17 NPV: Migraine: 92; TTH: 88; headache on more than 15 days: 99; probable MOH: 99
HARDSHIP questionnaire		Kukava M, et al. (2007) (29)	Georgia	Random sample from population survey (n = 186)	Neurological assessment by headache neurologist specialist	Sensitivity: Migraine: 75; TTH: 79; migraine plus TTH: 62 Specificity: Migraine: 96; TTH: 86; migraine plus TTH: 84 PPV: Migraine: 89; TTH: 80; migraine plus TTH: 40 NPV: Migraine: 89; TTH: 85; migraine plus TTH: 91
HARDSHIP questionnaire		Rao G, et al. (2012) (41)	India	Random sample of participants taking part in a household survey (n = 381)	Clinical assessment based on ICHD-II criteria by headache expert	Sensitivity: Any headache: 88 (83–91); Migraine: 63 (52–72); TTH: 57 (48–65); CH: 57 (48–65) Specificity: Any headache: 81 (74–87); migraine: 85 (81–89); TTH: 81 (76–86); CH: 82 (76–86) PPV: Any headache: 89 (84–92); migraine: 55 (45–65); TTH: 61 (52–69); CH: 61 (52–69) NPV: Any headache: 80 (73–86); migraine: 89 (85–92); TTH: 79 (74–84); CH: 79 (74–85)
Italian ICHD-II based questionnaire: 76 questions to diagnose most common primary headache types: migraine (with and without aura), TTH, and probable MOH. Intended for use by a doctor in epidemiological studies	Diagnostic	Abrignani G, et al. (2011) (11)	Italy	Consecutive patients referred for the first time to a headache centre (n = 50)	Neurological examination and assessment by headache specialist	Sensitivity: Migraine with aura: 100; migraine without aura: 100; TTH: 100; TTH subgroup: 2.3 66.6; probable MOH: 100 Specificity: Migraine with aura 93.3 (86–100); migraine without aura: 100; TTH: 100; TTH subgroup: 2.3 100; probable MOH: 100
Headache questions as part of HUNT 3 study: 14 self-completed items including pain intensity, duration and accompanying symptoms, plus over-the-counter drugs taken for headache, to identify chronic headache, MOH, TTH, migraine (with and without aura)	Diagnostic	Hagen K, et al. (2010) (23)	Norway	Random sample of participants who had completed a general health survey in HUNT 3 study, Norway (n = 297)	Semi-structured face-to-face interview by headache trained neurologist including clinical examination when indicated and classified in accordance with ICHD-II criteria and revised edition for MOH	Sensitivity: TTH: 96 (94–98); migraine: 51 (45–57); MOH: 75 (70–80); CH: 69 (62–74) Specificity: TTH: 69 (63–75); migraine 95 (92–98); MOH: 100 (99–100); CH: 99 (98–100)
Short self-completed questionnaire based on IHS criteria: Questions on headache frequency, duration, location, character of pain, intensity, accompanying symptoms, influence on work/activity ability, to identify: migraine, TTH, or “other types of headache”	Diagnostic	Rasmussen B, et al. (1991) (42)	Denmark	Participants who had completed a general health survey focusing on headache disorders (n = 712)	Standardised structured headache interview (using the same questions as questionnaire, plus additional ones to probe on individual basis) plus complete neurological examination by neurologist	Sensitivity: Migraine: 51; episodic TTH: 43; chronic TTH: 14 Specificity: Migraine: 92; episodic TTH: 96; chronic TTH: 100 PPV: Migraine: 50; episodic TTH: 95; chronic TTH: 100 NPV: Migraine: 93; episodic TTH: 46; chronic TTH: 97
The Brief Headache Screen (BHS): Short self-completed questionnaire – frequency of severe (disabling) headache, other (mild) headache and use of symptomatic medication to generate diagnoses of migraine, daily headaches and medication overuse	Screening	Maizels M and Burchette R (2003) (34)	USA	Three populations: 1. patients seen in an emergency department with primary headaches; 2. patients from a family practice, recruited from a sign in the waiting area; 3. patients seen at a headache clinic (n = 399)	Interviewed using the SDMQ (Tom et al. 1994) based on IHS criteria to diagnose migraine, asked about medication use, and then classified according to study protocol	Sensitivity: CH with migraine: 93; MOH: 86; daily headache syndromes: 94 Specificity: CH with migraine: 63; MOH: 79; daily headache syndromes: 54
German Language Questionnaire: For screening migraine, TTH and TAC. Based on ICHD-II with 20 self-completed yes/no response items. Subjects interviewed further about number of days with different headache types and number of days of acute pain or migraine	Screening	Fritsche G, et al. (2007) (20)	Germany	Consecutive patients seen in an outpatient headache clinic, plus patients with trigeminal autonomic cephalgias (TAC) recruited from same clinic, plus healthy subjects without headache (n = 278)	Face-to-face interview by headache expert neurologist, symptomatic headaches ruled out by clinical examination, Doppler and duplex sonography and computer tomography and MRI when necessary	Sensitivity: Migraine: 73.2 (63.2–81.7); TTH: 85 (73.4–92.9); TAC: 63.3 (52.9–72.7): migraine/TTH: 62.1 (42.3–79.3) Specificity: Migraine: 96.1 (92.2–98.4); TTH: 98.2 (95.4–99.5); TAC: 98.8 (96–99.8); migraine/TTH: 97.8 (94.9–99.3) PPV: Migraine: 91 (82.4–96.3); TTH: 92.7 (82.4–97.9); TAC 96.9 (89.2–99.6); migraine/TTH: 78.3 (56.3–92.5) NPV: Migraine: 87 (81.5–91.3); TTH: 95.9 (92.5–98.1); TAC: 83.2 (77.5–87.9); migraine/TTH 95.3 (91.8–97.6
*One headache type*						
*Cluster headache*						
Brief Self-Administered Questionnaire for Cluster Headache Screening: Two questions with yes/no responses on attack duration ( < 180 minutes if untreated) and conjunctival injection and/or lacrimation.	Screening	Dousset V, et al. (2009) (17)	France	Consecutive patients with a history of episodic or chronic cluster headache or migraine with or without aura seen in headache centre (n = 96)	Neurological examination by headache specialist based on ICHD-II	Sensitivity: 81.1 Specificity: 100 PPV: 100 NPV: 89.4
Questionnaire for the Detection of Cluster Headache: Based on ICHD-II, 16 self- completed questions validated, best discriminatory pattern: unilaterality of pain and presence of five of seven features: Pain severity and location, duration < 3 to 4 hours, frequency and daily reoccurrence of attacks, rhinorrhoea and restlessness	Screening	Torelli P, et al. (2005) (46)	Italy	Consecutive patients seen at headache centre, plus sample of patients with chronic cluster headache attending in previous 2 years (n = 71)	Neurological examination by headache specialist, and if needed, additional instrumental tests. Initially used 1988 IHS diagnostic criteria but updated after ICHD-II published	Reported here for “best discriminatory pattern of questions” Sensitivity: 100 Specificity: 95.1 PPV: 93.8 NPV: 100
*Probable Medication Overuse Headache*						
Brief self-completed screening tool for the diagnosis of probable medication over-use headache (pMOH): Four questions adapted from ICHD-II criteria: 1. Do you have headaches for more than 15 days/month? 2. Do you take treatment for attacks more than 10 days per month? 3. Is it for more than 3 months? 4. Is drug intake regular?	Screening	Dousset V, et al. (2013) (16)	France	Consecutive headache patients identified by their GP as probable MOH and seen at a headache clinic for the first time. All primary and other secondary headaches excluded (n = 77)	Clinical diagnosis by headache specialist, based on the second edition of the ICHD-II.	Sensitivity: 81 Specificity: 100 PPV: 100 NPV: 81.4 Question 3 was removed from the analysis because 90.5% of participants responded yes. The results reported here are for questions 1, 2 and 4
*Migraine*						
Asian Migraine Criteria (AMC): Face-to-face seven item questionnaire: Unilateral location, throbbing quality, nausea and/or vomiting, photophobia and/or sonophobia, osmophobia, family history of migraine and aura	Screening	Ghandehari K, et al. (2012) (22)	Iran	Consecutive adults attending a headache clinic over a 6-month period. Patients with probable diagnosis of migraine based on ICHD-II were excluded (n = 350)	History based on the ICHD-II taken by headache specialist	Sensitivity: 99.3 Specificity: 84.48 PPV: 96.93 NPV: 96.08
3-Question Headache Screen: 1. Do you have recurrent headaches that interfere with work, family, or social functions? 2. Do your headaches last at least 4 hours? 3. Have you had new or different headaches in the past 6 months?	Screening	Cady R, et al. (2004) (13)	USA	Recruited from private physicians' offices. Patients selected if they satisfied 1 of 3 migraine diagnostic criteria: IHS criteria for migraine (with or without aura), investigators clinical impression, or self-report of recurring disabling headaches (n = 3014)	1) IHS criteria; 2) clinical impression based on history and investigator's clinical experience or 3) recurring headaches based on self-reported or expert questioning by primary care physicians and neurologists	Overall sensitivity: 77%
ID Migraine: Three self-completed questions on disability, nausea and photophobia	Screening	Lipton RB, et al. (2003) (32)	USA	Primary care patients reporting ≥ 2 headaches in the previous 3 months that had limited their ability to work, study, or enjoy life or that they wish to consult a health professional about. After 1/3 recruitment completed added criteria that patients were excluded if they had a previous diagnosis of headache (n = 443)	Neurological history and examination (including additional diagnostic tests in applicable) and an IHS based semi-structured interview by headache specialist. Computer-based algorithm was run on the IHS criteria and compared with clinician diagnosis	Sensitivity: 81 (95% CI, 77–85) Specificity: 75 (95% CI, 64–84) PPV: 93.3 (95% CI, 89.9–95.8)
ID Migraine: Used here in different settings, and already validated in Turkish	Screening	Ertas M, et al. (2009) (19)	Turkey	Patients presenting at ophthalmology clinics (OC), ENT clinics (ENTC) or neurology clinics (NC) (n = 1021)	Examination and interview by a neurologist using a symptom checklist based on IHS criteria and assigned to a clinical diagnosis of migraine, TTH or other headaches	Sensitivity: NC: 87.9; ENTC: 86.6; OC: 79.9 Specificity NC: 74; ENTC: 74.4; OC: 76 PPV: NC: 86; ENTC: 80; OC: 86 NPV: NC: 76; ENTC: 83; OC: 67
Simple screening questionnaire: Four self-complete items: 1. Have you ever had migraine?; 2. Have you ever had severe headache accompanied by nausea?; 3. Have you ever had severe headache accompanied by hypersensitivity to sound and light?; 4. Have you ever had visual disturbances lasting 5–60 minutes followed by headache?	Screening	Gervil M, et al. (1998) (21)	Denmark	Twins who answered yes to questions on headache in a health questionnaire were eligible (i.e. at least one of the twins had headache and both twins were then included in the validation study) (n = 2035)	A semi-structured interview designed for diagnosing headache disorders, with special emphasis on migraine and TTH (12 questions) by a doctor trained in headache diagnosis	Sensitivity: 85 Specificity: 81 PPV: 49 NPV: 86 The sensitivity, specificity and predictive values were calculated on extrapolated data, as only a proportion of those that answered “no” to the four questions were interviewed.
Nine item self-completed questionnaire including items from ID Migraine. Logistic regression used to determine which combination of items for highest validity (same three as Lipton)	Screening	Kim S and Kim C (2006) (27)	Korea	Patients seen in Temporomandibular joint and Orofacial Pain clinic reporting ≥ two headaches in the previous 3 months who wished to consult about their headaches or reported that the headache interfered with their lives (n = 176)	Semi-structured diagnostic questionnaire based on IHS criteria and examination by headache specialist	Sensitivity: 58 (95% CI 45–72) Specificity: 98 (95% CI 76–100) PPV: 94 (95% CI 86–100) Nausea, photophobia and headache-related disability showed the highest individual sensitivity and reported here
*Migraine Screen Questionnaire (MS-Q*): Five-item questionnaire based on diagnostic criteria from IHS: Frequency and intensity; duration of > 4 hours; nausea; sensitivity to light/noise and headache related disability.	Screening	Lainez M, et al. (2005) (30)	Spain	Patients from a headache clinic, half with diagnosis of migraine according to neurologist evaluation and IHS criteria, half without migraine (n = 140)	Neurologist assessed migraine according to IHS criteria	Sensitivity: 93 (95% CI, 87–99) Specificity: 81 (95% CI, 72–91) PPV: 83 (CI 75– 91) NPV: 92 (CI, 85–99)
MS-Q: Five self-completed questions requiring yes/no response (as above). Scored ≥ 4 suspected migraine	Screening	Lainez M, et al. (2010) (31)		Consecutive patients attending primary care centres regardless of reason (n = 9346)	Primary care doctors diagnosed migraine according to their clinical judgement and IHS diagnostic criteria	Sensitivity: 82 (81–84) Specificity: 97 (97–97) PPV: 90 (89–91) NPV: 94 (94–95)
Migraine Assessment Tool (MAT): Eight question face-to-face interview asked verbatim	Screening	Marcus D, et al. (2004) (36)	USA	Community sample with a history of headache, recruited through advertisements (n = 80)	Headache diagnostic evaluation including history and general medical, neurological examination using IHS criteria by neurologist	Sensitivity: 89 Specificity: 79 PPV: 85 NPV: 84
A self-completed questionnaire and algorithm based on modified IHS criteria A simple self-report questionnaire for migraine	Screening	Michel P, et al. (1993) (37) Michel P, et al. (1993) (38)	France	1. consecutive outpatients from headache clinics (n = 171); 2. employees of a company consecutively detected as headache sufferers in their annual check-ups (n = 96); 3. employees of a company who reported suffering from headaches at least once every three months during their annual check-ups (n = 166)	Classified by senior neurologist into migraine or non-migraine Interview by senior neurologist specialised in the diagnosis and treatment of migraine	*Sensitivity* Sample 1: 97.8 (definite migraine); Sample 2: 94.9; Sample 1: 95.7–99.9 (possible migraine); sample 2: 90.6–99.2 Specificity: Sample 1: 52.9 (definite migraine); Sample 2: 78.4; Sample 1: 45.4–60.4 (possible migraine); Sample 2: 70.2–86.6 Sensitivity: 44 (95% CI 34.6–53.4) Specificity: 92.7 (95% CI 84.6–100)
ID migraine (already validated in Italian by Brighina F, 2005)	Screening	Mostardini C (2009) (39)	Italy	Patients diagnosed with primary headache in an emergency department then assessed in a headache centre outpatient clinic within 48 hours of discharge (n = 245)	Diagnosis by headache specialist according to the ICHD-II criteria using clinical data that had been collected from the emergency department	Sensitivity: 94 Specificity: 83 PPV: 99 NPV: 31 False positives for secondary headaches indicated that patients with cluster headache in particular answered positive to all three questions
Three question “decision tree” on headache frequency, laterality and impact on functioning	Screening	Pryse-Phillips W, et al. (2002) (40)	Canada	Participants selected by a neurologist with clinically definite migraine, TTH or other headache (n = 100)	Assessment by neurologist	Sensitivity: 86 Specificity: 73 PPV: 96 NPV: 38
Patient migraine questionnaire: Self-completed with support when needed of 18 items including: Migraine history, duration and severity of headache, social disability, headache symptoms and VAS for pain	Screening	Wang S, et al. (2008) (47)	Taiwan	Patients with chief complaint of headache attending neurology clinic for the first time. Patients with headaches ≥ 15 days in one month excluded (n = 755)	Diagnosis of migraine by doctor using 12-item Physician's Core Screening Questionnaire and interpreted using ICHD-II criteria-based computer algorithm	Sensitivity: 73 Specificity: 82 PPV: 91 Report for each item best validity for three item combination when two of three items present: Nausea/vomiting, photophobia and moderate or severe headache (reported here). Authors comment why three items different to ID migraine – probably cultural, as less likely to report headache disability
Single item migraine screening test: 1. Self-reported bothersome headache question; 2. stripe pattern hypersensitivity	Screening	Yuan H, et al. (2015) (48)	USA	Random sample of patients and their partners attending routine check-ups at an Obstetrics and Gynaecology clinic (n = 254)	A brief structured interview to assess headache frequency, severity, duration, associated symptoms, disability, family history, migraine disability (MIDAS). Diagnosis made retrospectively using the ICHD-III beta by researcher	1.Self-reported bothersome headache question Sensitivity: 82 Specificity: 85 PPV: 81 NPV: 86 2 Stripe pattern hypersensitivity Sensitivity: 44 Specificity: 80 PPV: 64 NPV: 65
*Migraine with and without aura*						
Headache questionnaire included in Nord-Trondelag Health Survey (HUNT): 13 self-completed questions based on IHS criteria for migraine	Classification	Hagen K, et al. (2000) (24)	Norway	Random sample of participants who had completed a general health questionnaire which included questions on headache (n = 167)	Clinical semi-structured interview by doctor experienced in headache disorders	PPV: 100 NPV: 62
The Finnish Migraine-Specific Questionnaire: Based on the IHS, a self-completed questionnaire developed to diagnose migraine with and without aura in family studies Particular attention paid to characteristics of migraine aura, and patients asked to describe aura in their own words	Diagnostic	Kallela M, et al. (2001) (26)	Finland	Stage 1: Consecutive patients attending outpatient neurological clinic diagnosed with migraine in accordance with IHS criteria by a neurologist (n = 100); Stage 2: Members of migraine families (taking part in another study), with and without migraine (n = 94)	Stage 1: Assessment at outpatient neurological clinic and diagnosis according to IHS criteria by neurologist; Stage 2: Clinical telephone interview by neurologist with migraine diagnosed in accordance with IHS criteria	Sensitivity: Stage 1: MA: 88; MA+MO: 96; MO: 100; Stage 2: MA: 89; MA+MO: 83; MO: 93; no migraine: 100 Specificity: Stage 1: MA: 97; MA+MO: 96; MO: 100; Stage 2: MA: 95; MA+MO: 97; MO: 100; no migraine: 98 PPV: Stage 1: MA: 78; MA+MO: 98; MO: 100; Stage 2: MA: 85; MA+MO: 91; no migraine: 96 NPV: Stage 1: MA: 98; MA+MO: 93; MO: 100; Stage 2: MA: 97; MA+MO: 93; MA: 99; no migraine: 0
deCODE Migraine Questionnaire (DMQ3): To diagnose migraine with aura and migraine without aura according to ICHD-II. Fifty six self-completed questions, and patients asked to describe aura in their own words	Diagnostic	Kirchmann M, et al. (2006) (28)	Denmark	Random sample of patients with migraine with aura, migraine without aura, and controls from national patient register and headache clinics (n = 147)	Semi-structured telephone interview and diagnosis by trained doctor according to ICHD-II. Time lag between reference and index 2–3 years	Sensitivity: Overall 99 (97–100); MA: 77 (63–90); MO: 91 (81–100); MA+MO: 63 (48–78) Specificity: Overall: 86 (75–97); MA: 88 (82–94); MO: 93 (88–98); MA+MO: 92 (87–97)
Structured Migraine Interview (SMI): Brief self-completed (or face-to-face/telephone interview) 10-item questionnaire based on ICHD II criteria for diagnosing migraine with or without aura	Screening	Samaan Z, et al. (2010) (43)	UK	All patients registered at a migraine clinic were approached to take part and a random sample who responded were included in the validation exercise (n = 200)	Clinical diagnosis based on ICHD-II by headache specialist	Sensitivity: 87 Specificity: 58 PPV: 97 NPV: 26
University of California-San Diego (UCSD) Migraine Questionnaire: A short self-completed 10-item questionnaire, distinguishes between non-migrainous headache, migraine with and without aura	Classification	Tom T, et al. (1994) (45)	USA	Consecutive sample recruited from people referred to headache clinic, about half were there for an initial consultation, and half had been previously diagnosed with migraine (n = 50)	Evaluation by neurologist using IHS criteria	Sensitivity: Migraine: 92–94; migraine without aura: 100; migraine with aura: 71–81 Specificity: Migraine: 100; migraine without aura: 91–94; migraine with aura: 100 PPV: Migraine: 100; migraine without aura: 82–83; migraine with aura: 100 NPV: Migraine: 82–88; migraine without aura: 100; migraine with aura: 83–88 (range is for agreement of each type of reviewer)
*Aura*						
Visual Aura Rating Scale (VARS): Diagnostic rating scale related to visual aura symptoms and characteristics (i.e. location, scotoma, zig-zag lines, duration 5–10 mins, and whether it develops gradually). VARS is intended as a supplement to the ICHD-II	Diagnostic	Eriksen MK, et al. (2005) (18)	Denmark	Random sample of participants from hospital registers and neurology outpatient departments (n = 213)	A trained physician conducted a telephone interview to diagnose participants based on the ICHD-II	Sensitivity: Score of ≥ 5 91 Specificity: Score of ≥ 5 96 PPV: 24 NPV: 98
*Chronic migraine*						
Identify Chronic Migraine (ID-CM): 12-item online questionnaire, includes headache frequency, headache symptoms (photo and phonophobia, headache severity, and nausea), prescribed and OTC medication in previous month, how often headache interferes with activities and making plans in last month.	Classification	Lipton R, et al. (2016) (33)	USA	Participants selected from earlier psychometric validation exercise and CaMEO Study sample (n = 111)	Clinical experts using a Semi-Dtructured Diagnostic Interview for migraine (SSDI-D), heavily based on ICHD-III beta	Sensitivity: Migraine: 83.5; chronic migraine: 80.6 Specificity: 88.6 Migraine: 88.5; chronic migraine: 88.6 PPV: Migraine: 96; chronic migraine: 91.5 NPV: Migraine: 62.2; chronic migraine: 75
*Menstrual migraine*						
The Menstrual Migraine Assessment Tool (MMAT): Face-to-face or self-completed 3-item questionnaire. 1. Do you have headaches that are related to your period most months? 2. When the headaches are related to my period, they eventually become severe 3. When my headaches are related to my period, light bothers me more than when I don't have a headache	Screening	Tepper S, et al. (2008) (44)	USA	Consecutive patients attending a headache centre (n = 250)	Headache specialist diagnosis using ICHD-II criteria and headache diary data	Sensitivity: 94 Specificity: 74 PPV: 67 NPV: 95

ICHD: International Classification of Headache Disorders; TTH: tension type headache; NDPH: new daily persistent headache; MOH: medication overuse headache; CDH: chronic daily headache; TAC: trigeminal autonomic cephalalgia; CH: chronic headache; MA: migraine with aura; MO: migraine without aura.

The number of participants included in the validation studies ranges from 50 to 9346. The majority of the tools are questionnaires, self-completed (n = 17), completed face-to-face with a clinician or researcher (n = 8), and self-completed online questionnaires (n = 2). The three remaining tools are computerised decision support systems designed to assist clinical diagnosis. The number of items in the questionnaires range from short single item migraine screen tests (48) to a longer 76-item questionnaire (11) plus the complex computerised diagnostic tools (14,15,35).

The tools have been validated in headache clinic settings (n = 14) as part of general health or household surveys (n = 6), neurology clinics or departments (n = 5), primary care settings (n = 4), emergency care (n = 3), community (n = 2) and other settings such as ophthalmology departments, temporomandibular joint and orofacial pain clinics, and obstetrics and gynaecology clinics (n = 3), with some tools validated in more than one setting.

In most studies the reference standard used to validate the tool is a face-to-face neurological assessment by a headache specialist doctor based on the ICHD criteria used at the time of validation. Exceptions include where the reference standard is conducted by a headache nurse specialist experienced in headache diagnosis (35), primary care doctors trained to use IHS criteria for migraine (31) and researchers (48). When reported, the time interval between the index test and reference standard is generally short (conducted on the same day, or within four weeks) although the longest interval reported was between two to three years (28).

There is wide variation in the reporting of the psychometric results across studies, with some reporting an overall sensitivity and/or specificity only, others reporting results for particular headache types, and with some studies also reporting positive and negative predictive values. We have reported the psychometric results for each study in Table 1.

### Study quality

The quality assessment for risk of bias for the four QUADAS-2 domains: Patient selection, index test, reference standard, flow and timing, plus an overall quality assessment, are reported in Table 2. The overall risk of bias was low for five studies, low/medium for 16, medium for seven, medium/high for six studies and high for four studies.

**Table 3.** Common questions across migraine screening tools .

	Headache location	Headache frequency	Headache duration	Pain severity	Pain quality	Pain restricting activity	Pain made worse by physical activity	Nausea/ vomiting	Photophobia	Phonophobia	Visual disturbance
Cady R, et al. (2004) (13)			×			×					
Gervil M, et al. (1998) (21)				×				×	×	×	×
Ghandehari K, et al. (2012) (22)	×				×			×	×	×	
Lainez MJ, et al. (2005) (30)		×	×			×		×	×	×	
Lipton RB, et al. (2003) (32)						×		×	×		
Marcus DA, et al. (2004) (36)	×	×	×		×	×	×	×	×	×	×
Michel P, et al. (1993) (37)	×	×	×			×	×	×	×	×	
Pryse-Phillips W, et al. (2002) (40)	×	×				×					
Wang S. et al. (2008) (47)				×				×	×		

### Multiple headache types

We identified nine tools that diagnose or classify more than one headache type, three computerised decision support tools, and six questionnaires. The three computerised diagnostic tools support clinical diagnosis of the common episodic and chronic primary headache disorders migraine and TTH, trigeminal autonomic cephalalgia (TAC) and medication overuse headache (MOH) (14,15,35). The sensitivity of the computerised decision support tools appears to be good for most headache types, the authors report lower sensitivity for probable migraine and probable TTH (15) and new daily persistent headache (NDPH) (35). All three tools have been validated in headache clinic populations; overall risk of bias is lowest for the study by Dong et al. (2014) (15).

The six questionnaires all classify the common primary headache disorders, migraine and TTH, plus MOH. The 76-item Italian ICHD-II based questionnaire reports the highest sensitivity and specificity and has low risk of bias (11). The HARDSHIP questionnaire, developed as part of a Global Campaign against Headache initiative, has been the most extensively validated, in different languages and cultures, and is suitable to be administered by trained lay interviewers; examples of studies using the questionnaire are presented in Table 1. Sensitivity ranges from 63–85 and specificity 82–99 for migraine, and sensitivity 51–64 and specificity 51–64 for TTH across the four studies (India, China, Russia and Pakistan) (49).

In addition to the common primary headache disorders, migraine and TTH, one self-completed questionnaire also screens for trigeminal autonomic cephalalgia (20), validated in a headache clinic population; authors report sensitivity 63.3 (52.9–72.7) and specificity 98.8 (96–99.8).

### One headache type

#### Cluster headache

We identified two tools that screen for cluster headache, both self-completed questionnaires validated in headache clinic populations with low/medium risk of bias (17,46). The presence of five of seven features (pain severity and location, duration < 3 to 4 hours, frequency and daily reoccurrence of attacks, rhinorrhoea and restlessness) has high sensitivity, 100, and specificity of 95.1 (46).

#### Probable medication overuse headache

We found one tool for the screening of probable medication overuse headache (pMOH) (16), validated in a headache clinic setting with low/medium risk of bias. Based on ICHD-II criteria for pMOH, three items were found to have sensitivity of 81 and specificity of 100.

#### Migraine

We identified 18 tools designed to classify or screen for migraine: Migraine only (n = 10), migraine with and without aura (n = 5), aura only (n = 1), chronic migraine (n = 1) and menstrual migraine (n = 1).

The majority of the migraine tools are short self-completed screening questionnaires; where authors have published the tool, the most frequent items are shown in Table 3. Notably, there are no questions common to all tools, but questions to identify nausea and/or vomiting related to headache (n = 7) and photophobia (n = 7) are most frequent across the tools, followed by pain restricting activity (n = 6) and phonophobia (n = 5).

**Table 2.** Methodological quality of studies using an adapted version of QUADAS-2 .

Author	Consecutive or random sample of participants	Case- control design avoided	Inappropriate exclusions avoided	Could the selection of patients have introduced bias?	Index test results interpreted without knowledge of the results of the reference standard	Could the conduct or interpretation of the index test have introduced bias?	Is the reference standard likely to correctly classify the target condition?	Reference standard results interpreted without knowledge of the results of the index test?	Could the reference standard, its conduct, or its interpretation have introduced bias?	Was there an appropriate interval between index test(s) and reference standard?	Did all patients receive a reference standard?	Did patients receive the same reference standard?	Were all patients included in the analysis?	Could the patient flow have introduced bias?	Overall Quality Score (low, medium or high risk of bias)
Abrignani G, et al. (2011) (11)	Yes	Yes	Yes	Low	Yes	Low	Yes	Yes	Low	Yes	Yes	Yes	Yes	Low	Low
Ayzenberg I, et al. (2011) (12)	Unclear	Yes	Yes	High*	Yes	Low	Yes	Yes	Low	Yes	Yes	Yes	Yes	Low	Low/medium*
Cady R, et al. (2004) (13)	Unclear	Yes	No	High*	No	High	Unclear	No	High*	Unclear	Yes	No	Unclear	High*	High*
De Simone R, et al. (2007) (14)	Unclear	Yes	No	High*	Yes	Low	Yes	Yes	Low	Unclear	Yes	No	Yes	High*	Medium*
Dong Z, et al. (2014) (15)	Unclear	Yes	Unclear	High*	Yes	Low	Yes	Yes	Low	Unclear	Yes	Yes	Yes	Low	Low/medium*
Dousset V, et al. (2013) (16)	Yes	Yes	No	high	Yes	Low	Yes	Yes	Low	Yes	Yes	Yes	Yes	Low	Low/medium
Dousset V, et al. (2009) (17)	Yes	Yes	No	High	Yes	Low	Yes	Yes	Low	Yes	Yes	Yes	Yes	Low	Low/medium
Eriksen MK, et al. (2005) (18)	Yes	Yes	Yes	Low	No	High	Yes	No	High	Yes	Yes	Yes	Yes	Low	Medium
Ertas M, et al. (2009) (19)	Yes	Yes	No	High	Yes	Low	Yes	Yes	Low	Yes	Yes	Yes	Yes	Low	Low/medium
Fritsche G, et al. (2007) (20)	Unclear	No	Yes	High	Yes	Low	Yes	Yes	Low	Yes	Yes	Yes	Yes	Low	Low/medium*
Gervil M, et al. (1998) (21)	No	Yes	Yes	High	Yes	Low	Yes	Yes	Low	No	No	Yes	Yes	High	Medium/high
Ghandehari K, et al. (2012) (22)	Yes	Yes	No	High	Yes	Low	Yes	Unclear	High*	No	Yes	Yes	Yes	High	Medium/high*
Hagen K, et al. (2010) (23)	Yes	Yes	Yes	Low	Yes	Low	Yes	Yes	Low	No	Yes	Yes	Yes	High	Low/medium
Hagen H, et al. (2000) (24)	Yes	Yes	Yes	Low	Yes	Low	Yes	Yes	Low	No	Yes	Yes	Yes	High	Low/medium
Herekar A, et al. (2013) (25)	Yes	Yes	Yes	Low	Yes	Low	Yes	Yes	Low	Yes	Yes	Yes	Yes	Low	Low
Kallela M, et al. (2001) (26)	Yes	Yes	No	High	Yes	Low	Yes	Yes	Low	Yes	No	Yes	Yes	High	Medium
Kim SK and Kim C-Y (2006) (27)	Unclear	Yes	Yes	High*	Unclear	High*	Yes	Yes	Low	Yes	Yes	Yes	Yes	Low	Medium*
Kirchmann E, et al. (2006) (28)	Yes	No	No	High	No	High	Yes	Yes	Low	No	Yes	Yes	No	High	Medium/High
Kukava M, et al. (2007) (29)	Yes	Yes	Yes	Low	Yes	Low	Yes	Yes	Low	No	Yes	Yes	No	High	Low/medium
Lainez MJ (2005) (30)	Unclear	Yes	No	High*	Unclear	High*	Yes	Unclear	High*	Yes	Yes	Yes	Yes	Low	Medium/high*
Lainez MJ (2010) (31)	Yes	Yes	No	High	Unclear	High*	Yes	Unclear	High*	Yes	Yes	Yes	No	High	High*
Lipton R, et al. (2016) (33)	No	Yes	Unclear	High*	Yes	Low	Yes	Yes	Low	Yes	Yes	Yes	Yes	Low	Low/medium*
Lipton R, et al. (2003) (32)	Unclear	Yes	Yes	High*	Yes	Low	Yes	Yes	Low	Yes	Yes	Yes	No	High	Medium*
Maizels M, et al. (2003) (34)	Unclear	Yes	Yes	High*	Yes	Low	Yes	No	High	Unclear	Yes	No	Yes	High*	Medium/high*
Maizels M and Wolfe WJ (2008) (35)	No	Yes	Yes	High	Unclear	High*	Yes	Unclear	High*	Unclear	No	Yes	Yes	High*	High*
Marcus D, et al. (2004) (36)	Yes	Yes	Yes	Low	Yes	Low	Yes	Yes	Low	Yes	Yes	Yes	Yes	Low	Low
Michel P (1993) (37)	Yes	Yes	Yes	Low	Yes	Low	Yes	Yes	Low	Unclear	Yes	Yes	Yes	High	Low/medium*
Michel P (1993) (38)	Unclear	Yes	Yes	High*	Yes	Low	Yes	Yes	Low	Unclear	Yes	Yes	Yes	High*	Medium*
Mostardini C, et al. (2009) (39)	Unclear	Yes	Yes	High*	Yes	Low	Yes	Yes	Low	Yes	Yes	Yes	No	High	Medium*
Pryse-Phillips W, et al. (2002) (40)	Unclear	Yes	Unclear	High*	Unclear	High*	Unclear	Unclear	High*	Unclear	Unclear	Unclear	Unclear	High*	High*
Rao G (2012) (41)	Yes	Yes	Yes	Low	Yes	Low	Yes	Yes	Low	Yes	Yes	Yes	Yes	Low	Low
Rasmussen B, et al. (1991) (42)	Yes	Yes	Yes	Low	Yes	Low	Yes	Yes	Low	Unclear	Yes	Yes	Yes	High*	Low/medium*
Samaan Z, et al. (2010) (43)	Yes	Yes	Yes	Low	Yes	Low	Yes	Yes	Low	Unclear	Yes	Yes	No	High*	Low/medium*
Tepper SJ, et al. (2008) (44)	Yes	Yes	Yes	Low	Yes	Low	Yes	Yes	Low	Yes	Yes	Yes	Yes	Low	Low
Tom T (1994) (45)	Yes	Yes	No	High	Yes	Low	Yes	Yes	Low	Yes	Yes	Yes	Yes	Low	Low/medium
Torelli P (2005) (46)	Yes	Yes	No	High	Yes	Low	Yes	Yes	Low	Unclear	Yes	Yes	Yes	Low*	Low/medium*
Wang SJ, et al. (2008) (47)	Unclear	Yes	No	High*	Unclear	High*	Yes	Unclear	High*	Yes	Yes	Yes	Yes	Low	Medium/high*
Yuan H, et al. (2015) (48)	Yes	Yes	Yes	Low	Yes	Low	Unclear	No	High*	Yes	Yes	Yes	Yes	Low	Low/medium*

*indicates when the criteria used to judge risk of bias for the domain is unclear.

The most widely used and validated of the migraine tools is the ID Migraine, with three questions on headache-related disability, nausea and photophobia (32). A previous meta-analysis of 13 studies reports sensitivity of 84 (95% CI, 75–90) and specificity of 76 (95% CI 69–83) (50). ID Migraine has been validated in different languages (not reported here) and different settings, including ENT and ophthalmology, and temporomandibular joint and orofacial pain clinics. (19,27).

The seven item Asian Migraine Criteria (AMC) reports the highest sensitivity and specificity (99.3 and 84.4), but medium/high* risk of bias (22). The eight item Migraine Assessment Tool (MAT) reports good sensitivity and specificity (89 and 79) and low risk of bias.

Not surprisingly, the tools developed to identify migraine with or without aura, all self-completed questionnaires, tend to be longer, ranging from 10 questions to 56 questions. The shortest of these, the University of California-San Diego (UCSD) Migraine Questionnaire (45) reports sensitivity and specificity for migraine with aura (71–81 and 100), migraine without aura (100 and 91–94) with low/medium risk of bias. A longer questionnaire where participants are asked to describe aura in their own words (26) reports higher sensitivity and specificity for migraine without aura (93–100 and 100) and migraine with aura (88–89 and 95–97) but has medium risk of bias (26).

The Visual Aura Rating Scale (VARS) (18) is intended to supplement the ICHD-II and captures visual aura symptoms and characteristics (e.g. location, scotoma, ziz-zag lines, duration, and gradual development). Validated in a population already diagnosed with migraine with aura, sensitivity and specificity are high (91 and 96) and methodological quality, medium* risk of bias.

Identify Chronic Migraine (ID-CM) (51) is an online tool to help clinicians identify patients likely to have migraine and, in particular, chronic migraine. The authors report sensitivity and specificity for migraine (83.5 and 88.5) and for chronic migraine (80.6 and 88.6); risk of bias is low/medium.

## Discussion

The review identified many papers validating headache diagnostic classification tools. The large number of tools identified indicates the demand for tools that can be used to support the diagnosis of headache disorders clinically and/or allow the classification of headache disorders in research.

More than half of the papers (n = 21) were judged to have low or low/medium risk of bias for overall quality. The risk of bias was judged high most frequently for the QUADAS-2 domain “flow and timing”, which assesses the interval between collecting the index and reference standard, the number of participants receiving the same reference standard, and whether all participants were included in the analysis. The criterion used to judge the risk of bias was unclear for at least one domain in 23 papers and recorded as “high risk”, indicating that quality assessment often reflects bias in reporting rather than bias in the conduct of the study. Most of the tools identified in the review have been tested in one setting only, predominantly in headache clinic populations. Exceptions include the Hardship tool, which has been validated in different languages and cultures, and the ID-migraine, which has been validated extensively in different languages and settings.

The overriding purpose of the review was driven by our own research requirements; that is, to identify existing headache classification tools that can be used by a non-expert clinician in primary care to classify chronic headache disorders, and specifically to identify tools that allow the user to screen for primary headache disorders other than migraine and TTH, distinguish between chronic migraine and chronic TTH and identify MOH. We anticipated that such a tool could also support primary care clinicians in diagnosing and managing chronic headache disorders within primary care more effectively.

We identified six tools that allow the user to screen for primary headache disorders other than migraine and TTH; four to identify trigeminal autonomic cephalalgias (TACs) and two specifically for cluster headache. The three computerised diagnostic tools appear to perform well for identification of TACs, as does the longer of the cluster headache-specific questionnaires (46). Only one of the computerised diagnostic tools was designed and validated for use by a non-expert clinician, but to date has not been validated in primary care (15).

The HARDSHIP questionnaire is the most extensively validated of the tools that allows the distinction between chronic migraine and chronic TTH by a non-expert clinician; the questionnaire was not designed to identify other primary headache types. The Identify Chronic Migraine (ID-CM) tool helps clinicians to identify patients likely to have migraine and, in particular, chronic migraine, but does not allow the classification of other chronic headache types. More commonly, the tools identified in the review classify episodic rather than chronic headache, with 17 screening for episodic migraine. It is interesting to note that, where authors have published the tool, no question is common to all tools.

The review also identified a number of tools that classify medication overuse headache including a brief self-complete questionnaire adapted from ICHD-II for MOH with sensitivity of 81 and specificity of 100 (16).

We identified a large number of good quality studies, validating a wide range of different headache classification tools; the review provides a comprehensive evaluation and summary of tools that researchers and clinicians can use to classify headache disorders. However, we did not identify a tool that fully met our own research needs that has been validated in a primary care setting and could be used by a non-expert clinician in primary care to support the diagnosis of common chronic headache disorders and screen for primary headache disorders other than migraine and TTH. We propose that such a tool could potentially support more targeted referral to headache specialists and free up under-resourced neurology departments (1).

### Strengths

We searched for tools that diagnose, classify or screen for all headache types rather than restrict the search to tools for chronic headache only.

All data extractions and quality assessments were double coded independently by three reviewers and agreement checked; we used the established tool QUADAS-2 to assess the quality of studies.

### Limitations

Our search strategy used MeSH terms based on the search terms used to develop NICE headache guidelines to identify potential validation studies, but it is possible that some studies were missed. Reporting of psychometric results made it difficult to compare across studies.

## Conclusions

Diagnosis of chronic headache disorders can be challenging for non-expert clinicians. Depending on the clinical or research need, there are a number of adequate tools available that could be used in specific contexts. Nevertheless, there are currently no tools validated in primary care that can be used by non-expert clinicians to classify chronic headache disorders that also allow the user to screen for primary headaches other than migraine and TTH. The availability of such a tool could support primary care clinicians in diagnosing and managing chronic headache disorders within primary care, and allow more targeted referral to headache specialists.

## Key findings

- The review identified 30 tools designed to diagnose, classify or screen for headache disorders, nine for multiple headache types, and 21 for one headache type only.
- Depending on the clinical or research need, there are a number of adequate tools available that could be used in specific contexts.
- There are currently no tools validated in primary care that can be used by non-expert clinicians to classify chronic headache disorders that also allow the user to screen for primary headaches other than migraine and TTH.

## Note

*indicates when the criteria used to judge risk of bias for the domain was unclear.
